# Acoustic and Articulatory Visual Feedback in Classroom L2 Vowel Remediation

**DOI:** 10.1177/00238309231223736

**Published:** 2024-05-01

**Authors:** Tanja Kocjančič, Tomáš Bořil, Susanna Hofmann

**Affiliations:** Institute of Phonetics, Charles University, Czech Republic; Faculty of Education, University of Ljubljana, Slovenia; Institute of Phonetics, Charles University, Czech Republic; Scandinavian Studies, Charles University, Czech Republic

**Keywords:** L2 vowel remediation, real-time visual feedback, ultrasound tongue imaging, formants, Swedish

## Abstract

This paper presents L2 vowel remediation in a classroom setting via two real-time visual feedback methods: articulatory ultrasound tongue imaging, which shows tongue shape and position, and a newly developed acoustic formant analyzer, which visualizes a point correlating with the combined effect of tongue position and lip rounding in a vowel quadrilateral. Ten Czech students of the Swedish language participated in the study. Swedish vowel production is difficult for Czech speakers since the languages differ significantly in their vowel systems. The students selected the vowel targets on their own and practiced in two classroom groups, with six students receiving two ultrasound training lessons, followed by one acoustic, and four students receiving two acoustic lessons, followed by one ultrasound. Audio data were collected pre-training, after the two sessions employing the first visual feedback method, and at post-training, allowing measuring Euclidean distance among selected groups of vowels and observing the direction of change within the vowel quadrilateral as a result of practice. Perception tests were performed before and after training, revealing that most learners perceived selected vowels correctly already before the practice. The study showed that both feedback methods can be successfully applied to L2 classroom learning, and both lead to the improvement in the pronunciation of the selected vowels, as well as the Swedish vowel set as a whole. However, ultrasound tongue imaging seems to have an advantage as it resulted in a greater number of improved targets.

## 1 Introduction

Adult learners of a foreign language (L2) often experience difficulties with the perception and production of L2 speech sounds, leading to mishearings of L2 speech or being judged as making mispronunciations. This has been observed in L2 learners of different languages: Spanish and Italian speakers learning English (Flege et al., 1999), Mandarin speakers learning English (Chen et al., 2001), and Japanese speakers learning French (Kamiyama & Vaissière, 2009). Importantly, such difficulties can persist even when learners achieve high proficiency in other aspects of L2 (Lundell et al., 2014).

According to the most widely accepted L2 speech sound acquisition theories, the source of these difficulties lies in the perceptual (dis)similarity between L1 and L2 speech sound categories. The Speech Learning Model (Flege, 1995) claims that a new L2 phonetic category can be only formed if learners can perceive dissimilarity between native and L2 speech sounds. Similarly, the Perceptual Assimilation Model for L2 Learners (Best & Tyler, 2007) states that the acquisition of a minimal contrast in L2 depends on how perceptually (dis-)similar one or both elements of an L2 pair are from the native phonetic categories. Both theories agree that adequate perception of L2 categories is the key to adequate L2 production. Practically, this suggests that L2 learners can never be more successful in L2 speech sound production than they are in perception.

The primacy of speech perception in L2 speech sound learning is further extended to the L2 classrooms where explicit pronunciation instruction is usually not part of the curriculum (for a review, see Derwing, 2018). Typically, it is expected that L2 learners will pick up the correct production by listening to a teacher or a model recording and trying to mimic what they hear. Although different perceptual methods have been proposed to facilitate L2 speech sound acquisition, high variability phonetic training (HVPT), which employs speech sound identification task, has received the most research interest (for review, see Barriuso & Hayes-Harb, 2018; Thomson, 2018). HVPT has shown significant improvements in the perception and production of previously inadequately acquired L2 sounds and the retention of gains over time (Bradlow et al., 1999; Lambacher et al., 2005; Wong, 2012). However, at least one study has shown no improvement in production following HVPT (Thomson & Derwing, 2016). Moreover, the degree of improvement depended on the number of the learner’s L1 phonetic categories (Iverson & Evans, 2009), with more categories being beneficial, and on the learner’s perceptual abilities (Perrachione et al., 2011). It is thus not surprising that some learners do not benefit from perceptual training and display obvious mispronunciations even after years of L2 learning.

When pronunciation is explicitly taught during an L2 class, it typically includes an articulatory description of the target sounds. This method has received more attention in recent years and has been reported to positively contribute to L2 pronunciation (Aliaga-García & Mora, 2009; Arteaga, 2000; Derwing et al., 1997, 1998). However, learning via articulatory description can be hindered by the teacher’s lack of knowledge of the articulatory processes; difficulties with understanding one’s own articulatory shape, position, and movements; poor ability to describe the target shape, position, or movements; and to follow articulatory instructions. A review by Derwing and Munro (2005) reports on several studies showing that many teachers of L2 English received no training on how to teach pronunciation. In addition, even if the teachers give appropriate articulatory instructions, learners will likely not understand or follow them correctly. Difficulties with the execution of even simple instructions on tongue movements were demonstrated in an ultrasound study by Ouni (2014). The experiment included 24 participants who were asked to execute 12 simple movement instructions (e.g., move your tongue forward, place your tongue in a position for /a/ and move it upward). The results showed that none of the participants could successfully execute two repetitions of any of the 12 movement instructions. Again, these results would explain why some L2 learners do not improve pronunciation even after giving detailed articulatory descriptions.

### 1.1 Visual feedback in L2 pronunciation training

Recent years have seen a change in L2 pronunciation training toward including different methods that allow real-time visualization of articulatory movements. The methods can bypass the difficulties related to giving verbal instructions on the shape, position, or movement of individual articulators (e.g., tongue, which is mostly not visible during speech) and understanding and realizing such instructions. Visual feedback (VF) methods can be based on the visualization of acoustic or articulatory properties of speech (for review, see Bliss et al., 2018).

#### 1.1.1 Acoustic VF

Acoustic VF methods have been attested in several studies that reported improved L2 pronunciation. The methods include indirect visualization of articulations via waveforms and spectrograms (Olson, 2014a, 2014b; Quintana-Lara, 2014; Ruellot, 2011) or formant representations (Carey, 2004; Kartushina et al., 2015). Both methods are based on the acoustic signal, but they differ in how speech is presented and, consequently, which articulatory characteristics can be displayed and how easy it is to understand the feedback.

The main advantage of spectrogram VF is that spectrograms can display several acoustic characteristics: segment duration, formants, presence of closure or noise, voice onset time (VOT), voicing, and so on. As such, they can be used for training pronunciation of vowels and consonants. However, the studies employing spectrograms showed that to benefit from this type of visualization, the learners need to receive some training on reading and using spectrograms. However, this requires a time investment, typically unavailable during an L2 language learning course.

VF in the form of formant representations can be used for vowel training only. Here, based on the F1 and F2 measurements, vowels can be visualized in real time in a vowel quadrilateral related to the horizontal and vertical positions of the tongue body in the oral cavity (both dimensions are also affected by lip rounding; however, the simplification is made for the sake of pedagogical application). Such representation makes the values easier to understand and relate to the speaker’s tongue position during speech production training.

#### 1.1.2 Articulatory VF

Articulatory VF includes different methods that directly visualize tongue activity during speech: electropalatography (EPG) visualizes tongue–palate contact via the insertion of an artificial palate with embedded electrodes into the speaker’s mouth; ultrasound tongue imaging (UTI) visualizes the entire tongue surface; and electromagnetic articulography (EMA) employs small electrodes being glued to the tongue, lips, and face, which makes it possible to track their movement in real time and rebuild a visual representation of an entire tongue and its position relative to any other electrodes. These methods originate in clinical settings, and particularly the first two have been used for speech sound remediation in speech and language therapy practice for several years (see the review in Sugden et al., 2019). Because of its ease of usage (no complex setup as in EMA) and no additional cost per speaker (as with EPG palate), UTI is the most practical and best suited for application in an L2 learning environment.

**Figure 1.**
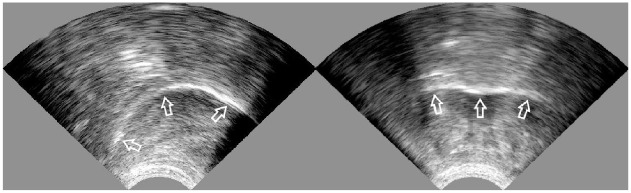
Sagittal (on the left; the front of the tongue is on the right side of the image) and coronal (on the right) ultrasound tongue images. The arrows mark the tongue surface at the bottom edge of the bright curve.

*Ultrasound* tongue *imaging*. UTI is a safe and non-invasive technique that, after placing the ultrasound probe under the speaker’s chin, images tongue surface in a sagittal and coronal view (Cleland et al., 2018; Stone, 2005; Wilson, 2014). The left image in Figure 1 shows the tongue surface (the lower edge of the bright curve marked by the arrows) in the midsagittal view (or view from the side of the face), with the front of the tongue being on the right side of the image, and the right image shows the tongue in the coronal view (or view from the front of the face). The images are obtained with an ultrasound machine, a standard medical device used to image internal soft tissue. The ultrasound probe placed under the speaker’s chin emits high-frequency ultrasound waves that travel straight upward through the soft tissue of the chin and tongue. Once they reach a boundary between two mediums of different densities, either tongue–bone in the case of tongue–palate contact or tongue–air in the case of no such contact, most of the waves are reflected back to the probe. After the detection and having the information on the elapsed time and the density of human tissue, the system calculates the point of reflection and marks it as a light point on the image. The final image shows a white curve, with its lower edge representing the tongue surface.

As evident in Figure 1, the image does not include any other anatomical structures or any representation of a static point in the oral cavity. Importantly, the image in sagittal view also cannot reliably include a raised tongue tip. Raising the tongue tip creates an air pocket below it, which can result in the emitted ultrasound waves being reflected at the boundary of the tissue-air pocket and not reaching the tongue tip.

When visualizing the tongue, it is crucial to position the probe appropriately to capture as much of the tongue as possible. In the sagittal view, the imaged tongue is limited by the jaw in the front and hyoid in the back, which create bone shadows on the image itself (Figure 1, left image)—ultrasound waves cannot travel through bone. In the coronal view, the image is limited by the jaw shadow on both sides (Figure 1, right image). The ideal probe position is such that the resulting image shows the shadows symmetrically. Because the ultrasound probe emits waves perpendicular to the curved shape of the probe, it is essential to note that the created image represents only the section of the tongue right above it. It also means that changing the exact probe location changes the imaged part of the tongue. For exact articulatory analysis, it is thus necessary to stabilize the probe relative to the head using different headsets or probe support systems. However, this is unnecessary for practical application in L2 pronunciation practice, and speakers can hold the probe under their chin.

Finally, we must be aware that the quality of the images is also speaker dependent. It relates mainly to the morphological characteristics, such as the structure of the tissue under the chin, which can cause greater dispersion of the emitted waves, and the size of the chin, which can limit the optimal probe position. Image quality further depends on the imaging depth setting, with greater depths resulting in poorer image quality. In practice, adults with larger heads typically need increased imaging depth since the distance from the probe to the imaging object (the tongue) is larger.

However, despite these limitations, the tongue surface image enables observation of tongue shape, position, and movement. In a sagittal view, we can independently observe the front, middle, and back of the tongue, as well as their coordination. On the other hand, the coronal view provides information about the position of the side of the tongue and the presence of the midline groove. The selection of the view depends on the practiced target. The sagittal view is sufficient for practicing vowels since it provides the necessary information about the tongue shape and vertical and horizontal tongue positions, as well as tongue shape and position for consonants. A coronal view is needed for highlighting and practicing particular articulatory elements of consonants (e.g., lateral bracing, lateralization, and the depth of the midline groove).

To perform UTI, relatively few materials are needed. Theoretically, almost any medical ultrasound machine can be used if it includes a probe with a head size suitable for positioning it under the chin, appropriate frequency, and scanning depth. Currently, there are two commercially available systems applicable for VF in speech sound remediation or pronunciation training that can be plugged into a laptop or tablet. Additional equipment consists of the ultrasound gel, which prevents the presence of air between the probe and the skin, and disinfectant probe wipes.

Until now, studies exploring UTI included a relatively small number of learners who practiced individual speech sounds during one to five 30- to 45-min individual training sessions (d’Apolito et al., 2017; Gick et al., 2008; Kocjančič Antolík et al., 2019; Roon et al., 2020). They all reported improvement in the target articulations and general user satisfaction with the method. However, offering several individual sessions is possible only in small-scale research studies and not when planning pronunciation training in a classroom. We are aware of only one study addressing the application of UTI to a classroom. In the study, seven native French speakers studying English received five sessions of 10 min of ultrasound practice within their regular university course, with the goal of improving the contrast of two vowel pairs: /iː - ɪ/ and /æ - ʌ/ (Kühnert & Pillot-Loiseau, 2022). Three participants showed an improved contrast of the vowel pair /iː - ɪ/ at the post-test, pointing to possible differences between speakers and between trained vowels.

### 1.2 The present study

An important difference between the acoustic and articulatory methods lies in their applicability to L2 classroom learning. Acoustic methods require a device to record an audio signal, perform the analysis, and display the result. Typically, this can be done on any computer, and the method can be part of any classroom learning with access to a computer lab or even via smartphones. On the other hand, UTI requires specific equipment, and the question remains of how it can be applied to the classroom setting.

The aims of the study were (1) to explore the practicality of using acoustic and articulatory VF methods in an L2 classroom, (2) to measure an individual change in vowel pronunciation because of using these methods and, to make the experiment possible, (III) to create a new tool for acoustic VF.

#### 1.2.1 Czech and Swedish vowels

Because the participants in the current study were Czech learners of Swedish who wanted to improve their production of Swedish vowels, it is important to highlight the main differences between the Czech and Swedish vowel systems.

**Figure 2.**
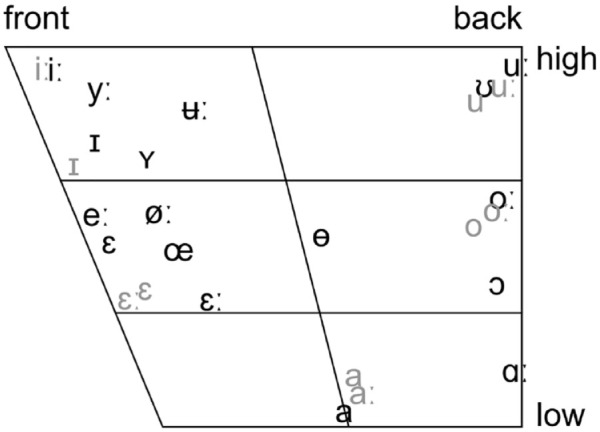
Swedish (black) and Czech (gray) vowels.

Czech and Swedish differ significantly in the number of monophthongal elements in their vowel sets. Czech contains ten vowels, while 17 are present in Swedish. Figure 2 displays Swedish vowels in black (Engstrand, 1999) and Czech vowels in gray (Šimáčková et al., 2012) (please note two phonetic realizations of the short closed front vowel: /i/ in Moravian Czech and /ɪ/ in Bohemian Czech).

The Czech vowel system contains five (six for Moravian Czech) distinct vowel categories: front high, front mid-high, central low, back mid-high, and back high. In contrast, the Swedish vowel system has additional vowels in the mid-open front, close central, mid-close central, mid-open back, and open back. Vowels in both languages are marked by quantity. All Czech vowels have short and long counterparts, while Swedish has nine long and eight short vowels. Both vowel systems include rounded and unrounded vowels. However, in Czech, only back vowels are rounded, while in Swedish, in addition to all back vowels, rounded vowels also appear in close central and mid-close central positions, as well as among the front vowels where they form rounded-unrounded pairs (/ʏ/–/ɪ/, /yː/–/iː/, /œ/–/ε/, and /øː/–/eː/).

Due to the predicted characteristics of L2 speech sound acquisition (Best & Tyler, 2007; Flege, 1995), it was expected that Czech learners of Swedish would display difficulties with Swedish vowel perception and production. Difficulties could be observed in forming contrast within front vowels and within back vowels, between front and central vowels, between open central and open back vowels, and between paired rounded and unrounded vowels. Another possible difficulty is vowel length contrast; however, because such a feature also exists in Czech, it is less likely to pose a problem. It has been shown earlier that learners of Swedish with short-long vowel distinction in L1 are more successful in acquiring quantity contrast in Swedish (McAllister et al., 2002). Because the study explored the application of acoustic and articulatory VF methods in L2 classroom, the pronunciation training focused on the horizontal and vertical distribution of the vowels within the oral space.

## 2 Methodology

### 2.1 Participants

Ten third-year university students (20–25 years old) of the Swedish language, all native Czech speakers from the Central Bohemian region, participated in the study. The students have not learned Swedish before starting university studies. According to their native Swedish teacher (third author), all had notable difficulties with the production of Swedish vowels; however, the exact number and type of difficult vowels varied across the students. The teacher also reported that students have more difficulties with correct tongue placement than with lip rounding. Because lip rounding is visible, it has been practiced previously, and students were aware of it. Because the aim of the study was to explore the practicality of using acoustic and articulatory VF methods in an L2 classroom, the students were asked to select, with the help of the teacher, the exact vowels that they would like to practice. The students selected between one to four vowels (Table 1). Finally, the teacher reported that the participants did not have difficulties with grapheme-phoneme correspondence in Swedish.

**Table 1.** Participants in the Ultrasound Tongue Imaging Followed by Formant Analysis (UTI-FA) and Formant Analysis Followed by Ultrasound Tongue Imaging (FA-UTI) Groups and the Target Vowels Selected by Each Student.

UTI-FA			FA-UTI		
P1	F	/ɛː, ɵ^a^/	P2	M	/iː, yː^a^/
P3	F	/yː^a^, uː^a^, ʉː^a^/	P7	F	/ɵ^a^, uː^a^/
P4	F	/oː^a^, ɛː, ɵ^a^/	P9	M	/ʊ^a^/
P5	F	/ʉː^a^, ɛː/	P10	M	/yː^a^, ɵ^a^, ʉː^a^, ʏ^a^/
P6	F	/yː^a^, oː^a^, ɛː/			
P8	M	/ɛ/			

a Marks rounded vowels.

For the purpose of this study, the students were divided into two groups (Table 1) corresponding to their usual language practice groups. The first group (UTI-FA) consisted of six students (five females and one male) who received two training sessions with articulatory VF (UTI), followed by one session with acoustic feedback (formant analysis). The second group (FA-UTI) also had six students who received two training sessions with acoustic feedback, followed by one session with articulatory feedback. However, two of the students from this group did not attend recording sessions, and only the remaining four (one female and three males) are presented here. Moreover, participant P7 did not attend the first training session, and P1 and P9 did not attend the second session and the second recording.

In addition, a female Swedish speaker who is teaching Swedish pronunciation has provided spoken data for a perception test.

### 2.2 Speech material

The data set included 30 words with minimal pairs or triplets covering all 17 Swedish monophthong vowels (Table 2). Of these, 23 words are monosyllabic (CVC), and 7 are bisyllabic (CVCV), with the target vowel in the first syllable. Target vowels were preceded by either a coronal (10 words) or non-lingual (20 words) consonant and followed by a coronal (28 words) or velar (2 words) consonant. The words were selected by the teacher based on the material used in the class and were all known to the students.

**Table 2.** Swedish Vowels and Corresponding Word List.

Vowel	Word
/iː/	sil
/yː/	myra, syl
/ɪ/	sill
/ʏ/	myrra, syll
/eː/	veka, hel
/øː/	lön, nöt
/ɛ/	vecka, hetta, häll
/œ/	lönn, nött
/ɛː/	häl
/ʉː/	ful
/ɵ/	full
/a/	tall, matt
/ɑː/	tal, mat
/ɔ/	håll, moll
/oː/	hål, mål
/ʊ/	mossa, bott
/uː/	mosa, bot

### 2.3 Training methods

This study used two different VF methods that allow visual real-time feedback on tongue position and shape. The focus of the training was on the correct tongue placement. However, if the learners omitted lip rounding, they were reminded to include it.

#### 2.3.1 Articulatory VF method

**Figure 3.**
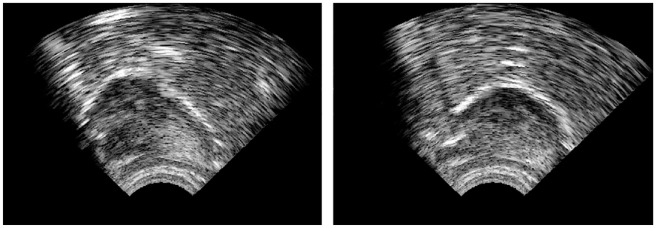
Ultrasound tongue images for /yː/ (on the left) and /uː/ (on the right). The front of the tongue is on the left side of both images.

The articulatory VF method employed real-time UTI with the Articulate Instruments Micro system (Articulate Instruments Ltd, 2012) on a laptop screen. The tongue was visualized in a midsagittal view, and the students held the probe under their chin by themselves. Figure 3 shows tongue images for /y:/ (on the left) and /u:/ (on the right) as viewed by the students. The two images clearly illustrate the difference between the high front tongue position for /yː/ and the high back tongue position for /uː/.

#### 2.3.2 Acoustic VF method

The acoustic VF method employed a real-time formant analyzer (available at https://fu.ff.cuni.cz/formants/) that displayed a position of a produced vowel in a vowel quadrilateral with F1 and F2 axes (a standard simplified 2D projection of the first two formants, since the F1 is related to vowel height and the F2 is related to the degree of backness, both also affected by lip rounding which was not visualized separately). It can run on a computer or a smartphone, the latter of which was used in pronunciation training. The formant analyzer has been developed as part of the study, and its full technical description is given in the Appendix.

**Figure 4.**
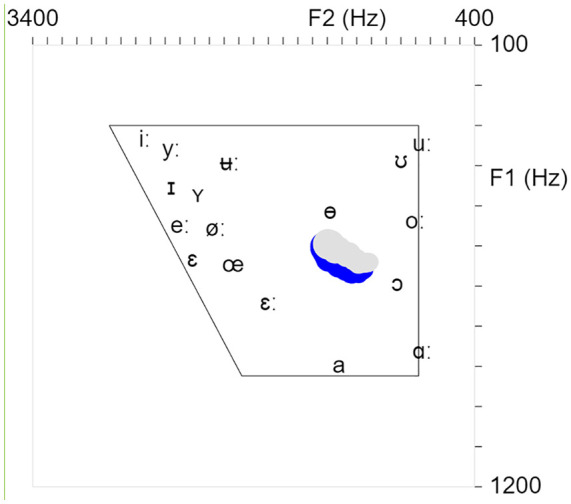
A screenshot of the formant analyzer with the trajectory toward the target vowel /ɵ/. The blue trajectory results from the main setting, and the gray from the alternative setting (see Appendix).

A screenshot of the formant analyzer, as viewed by the students, with the trajectory toward the target vowel /ɵ/ is represented in Figure 4. The blue and the gray trajectories relate to the main and the alternative analysis settings, respectively.

During the practice, the acoustic VF app constantly displayed the analysis (automatic formant detection) based on two settings: the main setup based on five formants and the alternative setup based on four formants with a significantly lower maximum frequency. In most situations, the main setup of the acoustic method produced stable trajectories. However, for back vowels and high vowels (e.g., [uː] or [oː]) with low F1 and F2 frequencies, the alternative approach setting produced a more stable trajectory, as it is less prone to integrate both low formants into one peak. The students were instructed to be aware of this behavior and to focus on the alternative approach trajectory for these vowels. Most of the time, both settings correlated well.

### 2.4 Training sessions

Pronunciation training was organized as a group (six students) classroom activity during students’ regularly scheduled weekly classes. During the class, each student received a 10-min individual VF training. A phonetician experienced with applying VF in L2 vowel remediation (first author) was providing instructions for the task to be practiced, giving additional information and, together with the Swedish teacher (third author), providing feedback based on the articulatory realization and perception.

At the beginning of the first session, the students were given a short description of the VF method they would be using. After that, the teacher demonstrated the production of Swedish vowels and highlighted their differences. Then, students took turns producing all Swedish vowels and compared their productions to the teacher’s. The students had to describe how their productions differed from the teacher’s. This task also allowed them to become familiar with tongue visualization (direct observation of the tongue in the case of UTI and position of the marker in the F1/F2 vowel quadrilateral). Once all the students completed this first task, they started with individual practice. Each student reported, with the help of the teacher, which vowels were the most difficult for them, and those were selected as practice targets. The vowels were practiced in isolation, and the practice lasted 10 min. Importantly, other students in the classroom (six in UTI-FA and four in FA-UTI) observed all their classmates’ individual training to become even more familiar with the visualization and to compare the VF with auditory perceived production. They were asked to think about the relation between the VF and the produced vowel, about their own tongue position when producing the same vowel, and about the change that the student has to make to improve vowel production.

The same feedback method was used in the second session. However, this time, the vowels were also practiced in nonwords with different consonant contexts and in real words.

In the third session, the feedback method was changed. Those who previously practiced with UTI were now using a formant analyzer and vice versa. Again, they were first given a short description of the method, and each student produced all Swedish vowels to become familiar with the method. Following this, they continued practicing the vowels in isolation, nonwords, and real words.

### 2.5 Data collection

#### 2.5.1 Production data

Audio recordings were obtained 1 week before the first training session, after the second session, and after the third one. P1 and P9 did not attend the second recording. Each student was presented with a written word list (Table 2) and was instructed to utter them once in the sentence “Jag säger __ igen.” (I say __ again). The same word list uttered in the same carrier sentence was additionally produced by the students’ teacher to obtain native speaker data, which were used in the perception tests.

#### 2.5.2 Perception data

Students performed two perception tests, 1 week before the first session and after the third session. The tests were given as identification tasks and consisted of two randomized repetitions of the word list (Table 2) extracted from the teacher’s recording. The isolated words were played via loudspeakers, and students had to write them down. It is important to note that the participants were familiar with the teacher’s pronunciation due to the teacher teaching them spoken Swedish, including pronunciation.

### 2.6 Data analysis

The practicality of using acoustic and articulatory VF methods in an L2 classroom was evaluated by verifying that all students received the planned amount of training and by monitoring that all students are engaged in the practice (as opposed to performing a different type of activity) and not only the one currently using a VF method.

The production and perception data were analyzed for each learner separately because of differences in the type and number of trained vowels and missing sessions.

#### 2.6.1 Production data

In all students’ recordings, the F1 and F2 formant values were chosen manually from these candidates by visual inspection of the spectrogram and listening procedure to avoid nasal formants mismatch and other possible errors of the automatic algorithm. The formant candidates were obtained by calculating a mean value in the middle third of the vowel duration in Praat (Boersma & Weenink, 2019) with three different settings of Burg method (25 ms window and 50 Hz pre-emphasis filter in common): (a) five formants with a maximum frequency of 5,500 Hz, a compromise between the default Praat settings for female speakers and the recommended settings for male speakers (Skarnitzl et al., 2015), (b) five formants with a maximum frequency of 3,000 Hz, and (c) 10 formants with a maximum frequency of 3000 Hz, where settings (b) and (c) were chosen to obtain a larger number of possible candidates for the subsequent expert manual selection in case of automatic extraction failure with setting (a). To quantify participants’ improvement in producing vowels, we measured Euclidean distances of F1/F2 formant values in equivalent rectangular bandwidth rate scale (ERBs; calculated in R [R Core Team, 2023] using the formula described by Moore & Glasberg, 1983) from the centroid of each group of vowels of interest (high front /iː, yː, ɪ, ʏ/, mid front /eː, øː, ε, εː, œ/, high back /ʊ, uː/, mid back /oː, ɔ/, low /a, ɑː/) and individually for the central vowels /ʉː/ within the group of all high vowels and for /ɵ/ within a group of all mid vowels. This allowed comparisons of the dispersion within the group between the first, second, and third recordings. For the first five groups (front high, front mod, back high, back mid, and back low), an increase in the Euclidean distance, resulting from greater dispersion of the included vowels, signaled an improvement in the production. For the two central vowels, a decreased measure resulted from a more central position of the target vowel within a group and, thus, an improvement in production.

The decision about the quality of change (improvement or decline) was made by visually comparing each learner’s vowel distribution in an F2/F1 plot to the standard vowel distribution of Swedish as presented by Engstrand (1999) in the Illustrations of the International Phonetic Alphabet. Please note that the published illustration does not provide information about any acoustic measures but serves as a description of the phonological inventory of a language (International Phonetic Association, 1999). The F1 and F2 measures obtained in the present study were similarly used to illustrate the position of the vowels in the vowel space and their relations to each other. The decision about the change was based on the comparison of the individual vowel position in the whole vowel space between pre- and post-training (comparing the first recording to the second, the second to the third and the first to the third). Specifically, the direction of change within the whole vowel system was noted.

#### 2.6.2 Perception data

Students’ responses were compared to the prompt items, and the number of errors in written responses was counted both before and after training.

## 3 Results

### 3.1 Production

**Figure 5.**
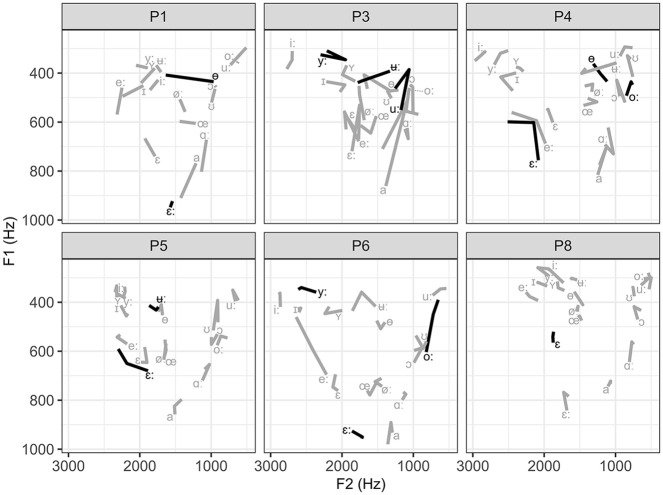
Change in the trained (black) and untrained (gray) vowels for learners in the UTI-FA group. The vowel symbol marks the F1 and F2 values pre-training, and the change in the line direction shows the values after two training sessions and the end of the line after the third training session.

**Figure 6.**
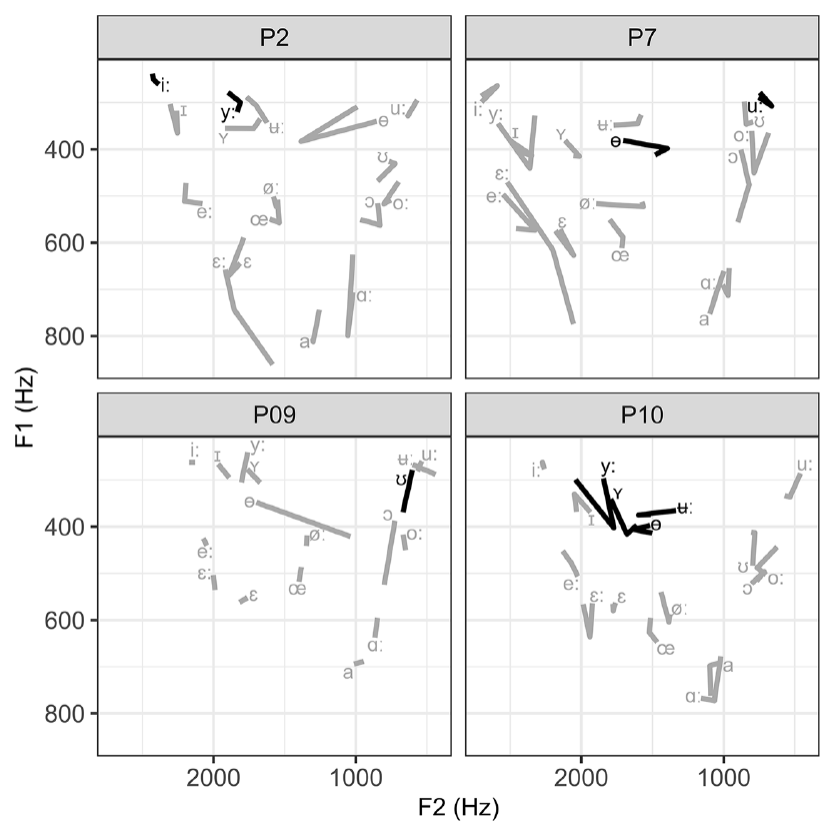
Change in the trained (black) and untrained (gray) vowels for learners in the FA-UTI group. The vowel symbol marks the F1 and F2 values pre-training, and the change in the line direction shows the values after two training sessions and the end of the line after the third training session.

Figure 5 shows the change in the trained (in black) and untrained (in gray) vowels for the UTI-FA learners and Figure 6 for the FA-UTI learners. Table 3 depicts Euclidean distances based on the vowel formants F1 and F2 in the three recording sessions and a three-way comparison between them (the first to the second, the second to the third, the first to the third).

**Table 3.** Euclidean Distance (in ERBs) Measured in Three Audio Recordings for Groups UTI-FA (Ultrasound Tongue Imaging Followed by Formant Analysis) and FA-UTI (Formant Analysis Followed by Ultrasound Tongue Imaging).

	UTI-FA						FA-UTI			
	P1^a^	P3	P4	P5	P6	P8	P2	P7^b^	P9^a^	P10
iː yː ɪ ʏ	0.57 – 0.81	1.18 **1.20** **1.58**	0.74 **0.87** **1.20**	0.23 **0.63** *0.59*	1.13 *0.93* **0.98**	0.60 **0.77** *0.62*	1.04 **1.39** *1.31*	0.85 **1.22** *1.09*	0.67 – 0.70	0.91 **1.31** *1.20*
eː øː ɛ ɛː œ	2.40 – 2.02	0.87 *0.84* 0.84	1.75 **1.98** **2.04**	0.89 **1.23** *1.17*	1.40 **1.72** **2.26**	2.05 *1.87* *1.73*	1.14 **1.47** **1.61**	1.34 *1.25* **1.53**	1.55 – 1.62	1.25 **1.28** 1.28
ʊ uː	1.60 – 2.58	0.53 **1.19** *0.16*	0.67 *0.35* **0.95**	1.65 *1.04* **1.53**	1.35 **2.06** **2.53**	0.60 **1.37** *1.36*	1.04 *0.92* **1.97**	0.49 **0.94** *0.54*	0.44 – 0.94	2.43 *1.48* *1.38*
a ɑː	0.71 – 1.04	1.44 *0.90* **1.20**	0.69 *0.38* **0.89**	1.36 **1.79** *1.21*	0.74 **0.87** **1.17**	1.32 **1.53** **1.64**	1.09 *0.83* **1.03**	0.46 *0.31* *0.15*	0.68 – 0.66	0.62 *0.39* **0.50**
oː ɔ	1.71 – 1.60	0.10 **0.73** *0.30*	0.40 **0.87** *0.68*	0.27 **0.58** **0.98**	0.77 **1.15** **2.08**	1.35 **1.66** *0.93*	0.43 *0.31* **1.33**	0.46 *0.24* **1.67**	0.37 – 0.90	0.52 *0.25* **0.89**
ɵ	2.99 – 2.68	1.71 *2.06* **1.69**	2.96 **1.95** *2.62*	2.10 *2.42* **1.84**	2.90 **1.97** **1.92**	2.21 **1.06** *1.81*	5.08 **2.66** *4.55*	1.51 *2.16* **1.87**	2.64 – 2.27	2.33 *2.48* **1.70**
ʉː	1.89 – 1.39	2.78 **0.52** **0.30**	4.90 **1.92** **1.60**	0.56 *1.16* *1.45*	0.80 *0.83* *0.81*	0.83 *1.33* *1.75*	0.83 *1.51* *1.68*	0.40 **0.26** *0.47*	5.86 – 8.03	0.44 *1.32* *0.76*

*Note.* The top number in each cell corresponds to the first recording, the middle one to the second, and the bottom one to the third. The first five rows depict groups of vowels of interest, where a mean Euclidean distance from their centroid is calculated; hence, an increased distance in the later recording marks a larger dispersion, i.e., an improvement. The last two rows of the table are focused on single vowels (/ɵ/ and /ʉː/), and hence, their Euclidean distance is measured from a centroid of closed-mid and open-mid front and back vowels (/eː, øː, ɛ, ɛː, œ, oː, ɔ/) in the case of /ɵ/ and a centroid of close front and back vowels (/iː, yː, ɪ, ʏ, ʊ, uː/) in the case of /ʉː/. In both cases, a decreased distance marks an improvement. Bold: improvement between the first and the second, or the second and the third recording. Italics: decline between the first and the second, or the second and the third recording. Gray: decline between the first and the third recordings.

a Missed the second session and the second recording.

b Missed the first training session.

Overall, the learners made positive changes in vowel production due to the VF pronunciation training. Comparing the results obtained in the first (pre-training) and the third (after the last training session) recording shows that one learner (P4) made positive changes in all seven categories evaluated with Euclidean distance, four learners in five, and five learners in four. In total, nine learners increased distance among the close front vowels (/iː, yː, ɪ, ʏ/), eight among the mid-back vowels (/oː, ɔ/), seven among mid-front (/eː, øː, ε, εː, œ/) and among the two close back vowels(/ʊ, uː/), and four among the open vowels (/a, ɑː/). Post-training, nine learners produced the vowel /ɵ/ more centrally among all the mid vowels, but only three learners produced /ʉː/ more centrally among the closed vowels. The vowels /ʊ, uː/ were often produced more posteriorly within individual learner’s vowel space at the third recording session, and /ɑː/ was positioned higher within the vowel space for most speakers.

The effect of the UTI-FA and FA-UTI method can be evaluated by comparing the results of the first recording to the second recording (after the two training sessions with either UTI-FA or FA-UTI; recall that learners P1 and P9 who did not attend the second recording are excluded from this analysis (see the Participants section). Among the closed front group, positive change was observed in 4/5 UTI-FA learners and 3/3 FA-UTI learners; among the mid-front vowels, 3/5 and 2/3; among close back group, 3/5 and 1/3; among open pair, 3/5 and 0/3; among mid back, 5/5 and 0/3, as well as in 3/5 and 1/3 for the production of vowel /ɵ/ and in 2/5 and 1/3 for vowel /ʉː/, respectively. These results suggest the advantage of the UTI-FA method for back vowels and closed central one and the advantage of the FA-UTI for the closed front vowels.

Because the learners each selected the exact vowels they wanted to practice, we will now present the change in these specific targets. The effect of training on the individual vowels is summarized in Table 4 by visually comparing the position of the target vowel in the whole vowel space and the direction of change within the vowel system between the first and the second, the second and the third, and the first and the third recording.

**Table 4.** Effect of Training on Individual Vowels After the Second (Compared to Pre-Training) and the Third (Compared to the Second) Sessions: Improvement (+), No Difference (=), Decline (–).

UTI-FA					FA-UTI				
		1^st^ – 2^nd^	2^nd^ – 3^rd^	1^st^ – 3^rd^			1^st^ – 2^nd^	2^nd^ – 3^rd^	1^st^ – 3^rd^
P1^a^	/ɛː/			=	P2	/iː/	=	=	=
	/ɵ/			+		/yː/	=	+	+
P3	/yː/	–	+	=	P7^b^	/ɵ/	+	=	+
	/uː/	+	–	+		/uː/	=	=	=
	/ʉː/	+	=	+	P9^a^	/ʊ/			+
P4	/oː/	+	=	+	P10	/yː/	–	+	+
	/ɛː/	+	+	+		/ɵ/	=	=	=
	/ɵ/	+	+	+		/ʉː/	+	=	+
P5	/ʉː/	=	=	=		/ʏ/	+	=	+
	/ɛː/	–	–	–					
P6	/yː/	+	=	+					
	/oː/	+	+	+					
	/ɛː/	–	=	–					
P8	/ɛ/	+	=	+					

a Missed the second training session and recording.

b Missed the first training session.

Learners practiced between one and four vowels. Out of 12 vowels (excluding P1 due to the missed second recording) practiced in the UTI-FA group, eight improved after the two articulatory sessions, three declined, and one showed no change. P5 was the only learner in this group who did not show improvement on the two practiced vowels due to the UTI-FA (however, this learner showed some positive changes in the organization of the vowel system, as seen in Table 3). After the additional acoustic training session, four targets improved even further, six showed no additional change, and two declined. Again, P5 was the only one showing no positive change on the two targets. Vowel /ʉː/ of P5 was further the only target that did not change because of the pronunciation training.

Learners in the FA-UTI group practiced eight vowels (excluding P9 due to the missed second recording). After two acoustic sessions, three vowels showed an improvement, four stayed the same, and one declined. This group also had one learner (P2) who made no improvement after the two FA training sessions. Importantly, only one training session was enough for P7 to improve one of the two trained vowels. After the additional articulatory session, two of the trained vowels showed improvement, while six did not change. This time it was P7 who did not benefit from the additional articulatory VF, while other learners showed improvement in at least one vowel. Vowels /iː/ of P2 and /uː/ of P7 did not show any change after the training sessions.

Comparing the productions of the first and the third recording gives a combined effect of training with two VF methods. Out of 23 practiced targets (all 10 learners combined), 15 were improved, six showed no change, and two declined.

In addition to the observed changes in the trained vowels, improvement was also noted on several untrained vowels and vowel contrasts in both experimental groups, as illustrated with the Euclidean distance measure mentioned earlier. The changes were learner-specific and do not allow any generalization. Within the UTI-FA group, P1 improved the /uː – oː/ and / ʉː – ʏ – yː – iː/ difference, P3 /eː – ε – εː/, /ɵ/ and /oː/, P4 /uː – ʊ/, /oː – ɔ/ and /ʏ – ɪ/, P5 /ʊ, oː, e/, P6 /eː, a, uː, ʏ/, /oː – ʊ/ and /eː – εː/, P8 /εː/, /uː – oː, /iː – yː/. Within FA-UTI group, P2 improved production of /uː/, /ε – εː/ and /oː – ɔ/, P7 /e – εː/ and /oː – ɔ/, P9 /ɵ/, /uː – ʊ/ and /oː – ɔ/, P10 /eː, øː/, /a – ɑ/ and /o – ʊ/.

### 3.2 Perception

Table 5 shows the number of correctly identified items (out of 60) for each participant. Two learners, P5 and P8, performed (almost) at the ceiling level at pre- and post-test. Except for P1, all learners correctly identified more items on the post-test than on the pre-test, with an increase of 4 (P3, P6, P2, P7, P9) or 7 (P4, P10) points. P2, P6, and P9 correctly identified all items on the post-test. P1 correctly identified three items less at the post-test than at the pre-test.

**Table 5.** Number of Correctly Identified Items (Out of 60) Per Participant in Groups UTI-FA (Ultrasound Tongue Imaging Followed by Formant Analysis ) and FA-UTI (Formant Analysis Followed by Ultrasound Tongue Imaging).

UTI-FA			FA-UTI		
	Pre	Post		Pre	Post
P1	59	56	P2	56	60
P3	54	58	P7	53	57
P4	47	54	P9	51	55
P5	58	59	P10	51	58
P6	56	60			
P8	59	60			

Out of 30 words, 15 were identified correctly by all learners on the pre-test and 18 on the post-test. The most errors were made on vowels /ɔ/ in “moll,” where eight learners wrote “mall” (not a word in Swedish) on the pre-test (two on the post-test), /ε/ in “häll” with six learners writing “hell” on the pre-test (three on the post-test), and /ʏ/ “syll” with four learners writing “sill” or “sylt” on the pre-test (none on the post-test). Interestingly, /ɔ/ in “håll” and /ε/ in “hetta” and “vecka” were identified correctly by everyone at both test points.

No mistakes either at pre- or post-test were made on vowels /a/ and /oː/. Vowels /ɵ, øː, œ, yː, ʏ/ were identified correctly by all learners after training, but not before training, while vowel /ɪ/ was identified correctly by everyone only at pre-test.

Comparing the vowels selected for training and the result of the perception tests revealed that most learners perceived all the “difficult” vowels correctly. After training, P2 improved the perception of words with the trained vowel /iː/ and P4 with /ɵ/. P4, however, also decreased the number of correctly identified words with /εː/, and P9 with /ʊ/.

## 4 Discussion

The first aim of the study was to test whether acoustic and articulatory VF methods can be used for pronunciation practice in an L2 classroom setting. By executing the planned VF training sessions, we have shown that both types of VF methods can be practically implemented into at least a small classroom of six learners. All students received the planned amount of individual training, and we did not observe any important time loss due to the switching of students. By asking the students to observe the training of their classmates, we assured their involvement in the articulatory practice throughout the lesson. In addition, we achieved the third aim by creating a new tool for acoustic VF and applying it to the L2 classroom.

Apart from one pilot study (Kühnert and Pillot-Loiseau, 2022), the UTI method has been previously used only in one-to-one sessions, partly because only one ultrasound system was available and partly because the pronunciation training demands many repetitions of the target articulation. Although it has been shown that even a 20-min practice with UTI improves the execution of simple articulatory instructions (Ouni, 2014) and that L2 learners can improve the production of individual speech sounds after a single 30-min session (Gick et al., 2008; Sisinni et al., 2016; Tateishi & Winters, 2013), it was not clear whether a very short UTI intervention, limited by the number of students and class duration, could have a similar effect. In the current study, each student practiced individually for about 10 min while the rest of the students were observing the practice. The results showed that the students in the UTI-FA group improved the target L2 vowel productions after only two training sessions or two times 10 min of individual practice. The practice time was even shorter than it was in the study by Kühnert and Pillot-Loiseau (2022), where the students also used UTI for 10 min per session but in five sessions.

Similar to the UTI-FA group, the FA-UTI group also showed improvement in vowel production after only two sessions. Importantly, the acoustic VF was significantly shorter than that in earlier studies reporting positive outcomes on training with a real-time visual representation of vowel formants. The participants in the study by Kartushina et al. (2015) received five 45-min training sessions and, in the study by Carey (2004), 5 hrs of practice.

The second aim of the study was to compare the effectiveness of the acoustic and articulatory VF methods. Due to the small number of participants and the difference in the number of practiced vowels, the results do not allow to formulate general conclusions. However, the UTI seems to have an advantage over the FA. The students improved 67% of vowels practiced in the two consecutive UTI sessions and 38% of those practiced in the two FA sessions. Moreover, the Euclidean distance improved for all attested vowel groups following the UTI practice but not for open and mid-back groups practiced with FA, suggesting clearer visualization of the production of these vowels with the UTI. Regardless of the method, students in both groups improved L2 vowel production and produced perceptually appropriate targets during the training (as judged by their teacher) after only a few practice attempts, as reported earlier in the study by Kocjančič Antolík et al. (2019). Both methods also caused positive changes in the entire Swedish vowel space, as seen in the improvement of untrained vowels or vowel contrasts. This was also observed for the three students (P1, P7, and P9) who participated only in two training sessions. We believe that the more general change was due to an increased awareness of the relation between tongue shape/position and produced vowels and increased awareness, and potentially control, of tongue movements in general. A beneficial effect of a short, 20-min observation of own tongue movements with UTI on tongue control has been shown earlier in a study by Ouni (2014). Observing their production in real time, via either method, additionally allowed students to improve the production of already known articulatory descriptions of Swedish vowels. For example, students P6 and P10 had shown improvements on /a/ and /ɑː/, two vowels that were not directly practiced in this study.

Furthermore, no method-specific advantage was noted after introducing a different method in the third session. The learners kept the gained change in the production or improved it further.

Finally, in an informal discussion at the end of the training session (and without seeing the results of the analysis presented here), the students reported that they were happy to use both methods and expressed interest in using them in the future. They agreed that the VF methods helped them better understand the tongue shape and position needed for specific vowels and increased the awareness of their own tongue shape, position, and movement. None of the learners had negative comments.

An important aspect of the study was the inclusion of active observation of the pronunciation practice of other students in the classroom. Such observation was expected to help the students better understand tongue articulation. During the training, some students silently practiced the same targets as those who worked with a VF method. In addition, the other students would occasionally remark on the correctness or the needed changes in articulation. In an informal discussion at the end of the training, the students agreed that watching other learners’ productions, as represented by acoustic or articulatory VF, helped them to better understand the characteristics of Swedish vowels, their own errors, and the changes they need to make to produce the vowels correctly. It is possible that observing the productions and training of others improved additionally the awareness of tongue movements and contributed to the noted improvement in the number of untrained vowels or vowel contrasts. Since the students selected individual training targets, each of them practiced between one and four Swedish vowels, but across them, they practiced seven different vowels in the UTI-FA group and seven in the FA-UTI group. Observing others thus exposed them to a greater number of vowels.

More practically, some differences between the articulatory and acoustic VF methods were noted. First, there is a difference in the amount of equipment and costs. To use the UTI method, one needs to have an ultrasound with a probe and software, a computer, and consumables such as ultrasound gel and disinfecting wipes. For the acoustic VF method, on the other hand, only a smartphone that can run an online app is needed. Second, following the initial explanation, the students understood the ultrasound images and could relate them to their tongue from the beginning of the training. Reading the visualization of formant was initially more challenging, and students needed a few attempts to relate their tongue to the position of the marker on the screen. In our experience, VF provided by UTI was also more appropriate for the training of close back vowels, as it clearly illustrated small differences in the tongue position, while the formant analysis made it possible to visualize the difference between rounded and unrounded vowels. Lip rounding is important for vowel discrimination in Swedish, and according to the teacher, the inclusion of sufficient lip rounding can be rather difficult for Czech learners.

Furthermore, the collected data allowed for observing the relationship between the learners’ speech production and perception. Following the two main theories of L2 learning (Best & Tyler, 2007; Flege, 1995), we expected that they would have problems with the same vowels in both domains, especially because both domains were attested with the same set of minimal pairs or triplets. This was, however, not the case. The learners had very few difficulties with the perception. Moreover, there was little overlap between the vowels in misidentified words and the “difficult” vowels chosen for pronunciation training. The results thus suggest that the effect of L1 phonology seems to be greater on L2 production than on L2 perception, possibly due to the difficulties in understanding one’s tongue movements (Ouni, 2014) and executing necessary new articulatory movements.

Finally, the study explored pronunciation training in a rather rare language pair, contributing to the needed diversity in this subject area.

### 4.1 Limitations and future directions

Although the current study applied VF methods to classroom L2 learning, the classes were still relatively small (six students per class), and the question remains whether the methods could be applied to larger classrooms. This has to be addressed in future research, possibly exploring the observation that students benefit from observing the VF pronunciation practice of others after the initial short practice with the same VF. This would further enable the inclusion of more learners as well as more balanced groups in terms of the number of participants and the type and number of practiced targets.

In addition, to fully describe post-practice changes in production, articulatory data (recorded with UTI) should be analyzed in a subset of participants. Although UTI data do not allow direct comparison of tongue images across different recording sessions (due to the impossibility of securing the same probe placement), the changes in L2 targets could be compared to the stable production of native speech sounds within each recording or by observing a change between a pair of L2 speech sounds to illustrate increased contrast (Kocjančič Antolík et al., 2019). The production data should also be collected in a more real-life setting (e.g., picture-story description or map task) to evaluate the transfer or practice gains to a conversation-like form and not only to isolated words in a carrier sentence. In addition, it would be informative to evaluate the long-term retention of training goals.

## 5 Conclusion

The aim of the current study was to explore the application of acoustic and articulatory VF methods in L2 classroom and to compare real-time UTI and formant VF for L2 vowel remediation. The results showed that Czech learners of Swedish improved in the production of trained and untrained vowels after a very short practice with either method, with a possible slight advantage of the UTI. Both methods also proved to be applicable to L2 classroom pronunciation training by combining short individual practice and active observation of other students’ practice, and both methods received high user satisfaction. Finally, the study highlighted the mismatch between the ability to perceive and produce L2 speech sounds.

## Appendix

The algorithm for the formant analyzer is implemented by the second author in HTML5 and JavaScript using Web Audio API. The input audio signal is sampled with a sample rate determined by a web browser (typically 44,100 Hz or 48,000 Hz) and sent in buffers of 1,024 samples. From this signal, the F0 frequency is estimated using the autocorrelation method via fast Fourier transform (FFT). For this purpose, the signal is stored in an array of length derived from the minimum duration of four periods of the minimal detectable frequency of 70 Hz (the length in samples is rounded up to the power of two). From the autocorrelation function, the first local maximum after the first zero crossing is found, and the corresponding frequency (if not larger than a maximum frequency of 600 Hz) is passed to a nonlinear filter remembering the last five values. If all five values are in the interval of 40 cents from the last inserted value, it is decided to be a new F0 estimate. If F0 is greater than 220 Hz, frequencies of vowel labels are switched to the female vocal tract (20% higher than male reference values). If F0 is lower than 175 Hz, the positions are switched to a male vocal tract. This decision introduces hysteresis leading to a stable behavior. The automatic estimation can be switched off, and the vocal tract may be chosen manually.

For each incoming buffer of the input signal, formant frequencies are estimated via two settings. The main setup finds five formants in the range of a maximum frequency of 5,500 Hz. The alternative approach finds four formants with a maximum frequency of 3,000 Hz, leading to a more stable recognition of high back vowels characterized by low frequencies of the first two formants. A mean value is subtracted from the buffer, and the buffer is then multiplied by the Hann window. Then, the signal is resampled to the sample rate equal to double of maximum frequency. The resampling is performed in the spectral domain by erasing a part of the spectra to the new range; the transfer between the time and spectral domain is implemented using Chirp Z-transform (Rabiner & Gold, 1975), which applies the next power-of-2 FFT method. The resampled signal is filtered via the pre-emphasis filter with the transfer function

(1)
$$H\left(z\right)=1-0.93z^{-1}$$

To find formant frequencies, the Burg method of linear predictive coding (LPC) (Anderson, 1978) (order equal to double the number of formants) estimates filter coefficients. Their roots are then calculated. For all roots with a non-negative imaginary part, argument *ϕ* and absolute value|*r*,| a frequency is calculated

(2)
$$f=\frac{\phi f_{s}}{2\pi }$$

where *f_s_* is the new sample rate. A bandwidth is (Snell & Milinazzo, 1993)

(2)
$$B=-\frac{f_{s}}{4\pi }\ln(|r|).$$

If the root-mean-square amplitude of the buffer exceeds a silence threshold and 90 < *f* < 5000, *B* < 40, and *B* / *f* < 0.2, the frequency (2) is accepted as a formant frequency. If two formants are found at least, and the first two lowest values are in the range of 100 to 1,200 Hz and 400 to 3,400 Hz, respectively, they are marked as F1 and F2. Both F1 and F2 values are filtered via independent moving average filters (order of 15) and are stored in a buffer, preserving the last 30 values for plotting a trajectory depicting the recent history in two-dimensional vowel space. The main and the alternative approach settings are plotted simultaneously with a color distinction.
